# Molecular Properties of Drugs Handled by Kidney OATs and Liver OATPs Revealed by Chemoinformatics and Machine Learning: Implications for Kidney and Liver Disease

**DOI:** 10.3390/pharmaceutics13101720

**Published:** 2021-10-18

**Authors:** Anisha K. Nigam, Anupam A. Ojha, Julia G. Li, Da Shi, Vibha Bhatnagar, Kabir B. Nigam, Ruben Abagyan, Sanjay K. Nigam

**Affiliations:** 1 Skaggs School of Pharmacy, University of California San Diego, La Jolla, CA 92093, USA; anishaknigam@gmail.com (A.K.N.); dsucsd@gmail.com (D.S.); 2 Department of Chemistry and Biochemistry, University of California San Diego, La Jolla, CA 92093, USA; aaojha@ucsd.edu; 3 Department of Biology, University of California San Diego, La Jolla, CA 92093, USA; li.julia.gao@gmail.com; 4 Department of Family and Preventative Medicine, University of California San Diego, La Jolla, CA 92093, USA; vbhatnag@ucsd.edu; 5 School of Medicine, Virginia Commonwealth University, Richmond, VA 23298, USA; nigamkb@vcu.edu; 6 Departments of Pediatrics and Medicine (Nephrology), University of California San Diego, La Jolla, CA 92093, USA

**Keywords:** artificial intelligence, machine learning, chronic kidney disease (CKD), organic anion transporter, xenobiotic transporter, remote sensing and signaling theory, nephrotoxicity, acute kidney injury (AKI), hepatic, biliary

## Abstract

In patients with liver or kidney disease, it is especially important to consider the routes of metabolism and elimination of small-molecule pharmaceuticals. Once in the blood, numerous drugs are taken up by the liver for metabolism and/or biliary elimination, or by the kidney for renal elimination. Many common drugs are organic anions. The major liver uptake transporters for organic anion drugs are organic anion transporter polypeptides (OATP1B1 or SLCO1B1; OATP1B3 or SLCO1B3), whereas in the kidney they are organic anion transporters (OAT1 or SLC22A6; OAT3 or SLC22A8). Since these particular OATPs are overwhelmingly found in the liver but not the kidney, and these OATs are overwhelmingly found in the kidney but not liver, it is possible to use chemoinformatics, machine learning (ML) and deep learning to analyze liver OATP-transported drugs versus kidney OAT-transported drugs. Our analysis of >30 quantitative physicochemical properties of OATP- and OAT-interacting drugs revealed eight properties that in combination, indicate a high propensity for interaction with “liver” transporters versus “kidney” ones based on machine learning (e.g., random forest, k-nearest neighbors) and deep-learning classification algorithms. Liver OATPs preferred drugs with greater hydrophobicity, higher complexity, and more ringed structures whereas kidney OATs preferred more polar drugs with more carboxyl groups. The results provide a strong molecular basis for tissue-specific targeting strategies, understanding drug–drug interactions as well as drug–metabolite interactions, and suggest a strategy for how drugs with comparable efficacy might be chosen in chronic liver or kidney disease (CKD) to minimize toxicity.

## 1. Introduction

A major issue in patients with impaired liver or kidney function is the route of elimination of prescribed drugs. Many common drugs such as beta-lactam antibiotics (e.g., penicillin), antivirals (e.g., tenofovir), analgesics (e.g., ibuprofen), statins used for hypercholesterolemia (e.g., pravastatin), antihypertensives (e.g., hydrochlorothiazide), and chemotherapeutics (e.g., methotrexate) are small organic anions [1,2,3,4,5,6]. Upon entering the blood through oral or parenteral routes, they are frequently taken up by the liver, where they may be modified by drug metabolizing enzymes (DMEs). The unmodified and modified drugs are generally eliminated into urine after kidney uptake or into the bile via the hepatobiliary system [7,8,9,10,11,12,13,14,15].

Although several hepatic and renal transporters can interact with organic anion drugs, four primary organic anion transporters involved in drug handling that have been initially highlighted by regulatory agencies are predominantly hepatic members of the OATP (SLCO) family and the predominantly renal members of the OAT (SLC22) family of solute carriers [16]. Specifically, these transporters are OATP1B1 (SLCO1B1) and OATP1B3 (SLCO1B3), both highly expressed on the basolateral (blood) surface of liver hepatocytes, and OAT1 (SLC22A6) and OAT3 (SLC22A8), both primarily expressed on the basolateral surface of kidney proximal tubule cells [7,13,17,18,19,20]. Apart from organic anion drugs, these transporters are also involved in the transport of metabolites, signaling molecules, nutrients, gut microbe products, and uremic toxins [21,22,23,24,25,26,27,28,29,30,31,32,33].

Transport assays indicate some drug ligand overlap between the liver OATPs and kidney OATs. Although certain organic anion drugs (e.g., methotrexate, pravastatin) can interact with at least one of the OATPs and one of the OATs at relatively high affinity (K_i_ ≈ 50 μM) [34,35], there are many drugs that have a high affinity for at least one of the OATPs but not one of the OATs, and vice versa. Here, we focus on this latter group of drugs that have a strong preference for at least one of the liver OATPs versus at least one of the kidney OATs. This is clinically important for understanding which of several existing drugs that can be used for a medical problem might be best suited to a patient with liver disease or kidney disease. From a drug design perspective, the type of analysis we are undertaking may be helpful for designing drugs that selectively target the liver or the kidney [27,36,37]. Furthermore, since OATPs and OATs have well-established roles in the transport of metabolites and signaling molecules (e.g., prostaglandins, bile acids, uric acid), understanding the physiochemical properties of drugs handled by the aforementioned OATPs and OATs and comparing them to the physiochemical properties of other drugs and endogenous metabolites handled by these transporters should aid in in silico drug–drug interaction (DDI) prediction and drug–metabolite interaction (DMI) prediction [7,16,38,39,40,41,42,43,44,45,46].

Here, we have curated a list of drugs reported to interact with at least OATP1B1 or OATP1B3 with high affinity (based on K_i_ values) while not having a reported high affinity for either OAT1 or OAT3. Similarly, we have curated another list for drugs reported to interact with high affinity with at least OAT1 or OAT3 but not OATP1B1 or OATP1B3. We then obtained or calculated over 30 chemoinformatic metrics (molecular properties) for these drugs. Using a variety of data visualization, statistical and informational methods, we narrowed down the key properties distinguishing “liver OATPs” versus “kidney OATs”. Eventually, we settled on eight molecular properties. Using a broad range of machine learning (e.g., k-nearest neighbors, random forest, decision tree, logistic regression) and deep-learning (neural network) approaches, we were able to use these molecular properties to classify OATPs versus OATs with high accuracy. Indeed, as high as >95% classification was obtained with certain methods. This very high classification accuracy based on only a relatively small set of molecular properties paves the way for new approaches to tissue targeting of small-molecule drugs, prediction of metabolic side effects, and tailoring drug therapy in the setting of organ injury or impairment (e.g., chronic kidney disease, acute renal injury, liver disease).

## 2. Materials and Methods

### 2.1. Curation of Drug Lists

A list was curated of drugs that interacted selectively with at least one OATP (OATP1B1 and/or OATP1B3), but not OAT1 or OAT3; a similar list was curated for drugs that interact with at least one OAT (OAT1 and/or OAT3), but not an OATP. The cut-off used was a measured or calculated K_i_ < 250 μM (Supplementary Materials Table S1). If a drug (e.g., pravastatin, methotrexate) had a K_i_ < 250 μM for an OATP and an OAT, it was not included to focus on features important to hepatic uptake by OATPs or renal uptake by OATs. Articles used for list curation are indicated in Supplementary Materials Table S2. Very often in the same article, there were data for both OATP1B1 and OATP1B3, or for OAT1 and OAT3. Rarely there were data for all four transporters in the same article. If high affinity of a drug for an OATP was reported in one article, but we could not find the affinity for an OAT in the literature, that drug was not included. It is also possible that we missed some relevant reports. However, we believe such situations are likely to be few, and given the robust results, unlikely to appreciably affect the classification metrics shown in the Results. In addition, we also note that the data are largely based on K_i_ values from cell-based *in vitro* systems, and whether these preferences hold in vivo remains to be verified in most cases.

### 2.2. Cheminformatics

2D chemical structures of drugs were imported and standardized (removing salts, explicit hydrogens and standardizing chemical groups) using various tools found in the computational environment of commercially available cheminformatics software package ICM Chemist (Molsoft). Once standardized, a set of 77 physicochemical properties for the chemicals were calculated. Complexity values for the chemicals were obtained from PubChem.

### 2.3. Defining Key Molecular Properties Distinguishing Kidney OATs vs. Liver OATPs

From a broader set of over 70 molecular properties, a smaller subset of properties was selected for classification using machine learning and deep learning. These structural and physicochemical properties were chosen in such a manner to identify critical features distinguishing drugs interacting uniquely with at least one OATP (OATP1B1 and/or OATP1B3) or at least one OAT (OAT1 and/or OAT3). An initial step was the elimination of highly correlated features by plotting the pairwise-correlation coefficients. For example, molecular volume was highly correlated with several other measures of molecular size or mass.

Furthermore, from a reduced set of more than 30 molecular properties (as described in Results), data visualizations (e.g., distributions, boxplots), measures of information gain, predictor screening (using Bootstrap/Random Forests in JMP Pro 15), principal components analysis, and FreeViz (Orange) were used to reduce the set of features to eight.

### 2.4. Machine Learning and Data Visualization with Orange

For interactive data analysis, data visualization, and machine learning, we used the Orange version 3.16 machine learning tools, which are largely based on the python library scikit-learn [47,48]. Data analysis methods included principal component analysis (PCA), FreeViz diagram, and application of several machine learning classification methodologies such as random forest, naïve bayes, decision trees, simple neural networks, k-nearest neighbors and logistic regression. The “leave-one-out” method was generally used for cross-validation. Other details are indicated in Results or in Figures. Orange default parameters were generally used for the purpose of analysis. However, we note that some of these parameters were varied when individual classification algorithms were performed using python.

### 2.5. Machine Learning and Data Visualization with Python Libraries

Although most of the data shown in the text comes from the Orange software package, data wrangling, data analysis, machine learning, and deep-learning codes were also written in Python 3.8 [49]. Python libraries for data analysis included pandas, NumPy, and SciPy. For data visualization, we used seaborn, matplotlib, pydotplus, and plotly. Machine learning and deep-learning codes were written using Keras, scikit-learn and TensorFlow. In general, similar analysis and results were obtained from independently written python scripts in a jupyter notebook as compared from those obtained with Orange.

### 2.6. Deep-Learning Classification

Analysis of critical features to classify drugs selectively interacting with liver OATPs (OATP1B1 and/or OATP1B3) versus kidney OATs (OAT1 and/or OAT3) has been achieved independently via built-in functions in Orange and with python scripts using scikit-learn, Keras and Tensorflow in jupyter notebooks. Multiple independent runs could classify these drugs with a very high accuracy exceeding 95%.

## 3. Results

### 3.1. Overview

Many commonly prescribed drugs are small organic anions. After entry into the circulation, they are often taken up into the liver (for metabolism by Phase 1 and Phase 2 drug metabolizing enzymes and/or biliary elimination) or the kidney (for elimination into the urine). In the case of liver, entry often occurs via the major multi-specific drug transporters: the organic anion transporting polypeptides, OATP1B1 (SLCO1B1) or OATP1B3 (SLCO1B3), grouped below as the main “liver” organic anion uptake (basolateral) transporters (“liver OATPs”). In the case of kidney, major multi-specific basolateral drug transporters are the organic anion transporters: OAT1 (SLC22A6) or OAT3 (SLC22A8), grouped below as the kidney transporters of drugs that are organic anions (“kidney OATs”). Because these particular OATPs have little if any expression in the mature kidney, and because these particular OATs have little if any expression in the mature liver, the “operational” groupings into liver and kidney transporters of (organic anion) drugs is appropriate for the aim at hand. Although low expression of OAT3 has been reported in fetal liver, in the adult liver there is minimal if any expression [18,23]. Although there are other transporters of organic anions expressed in both organs, their quantitative importance in in vivo handling of small organic anion drugs in humans is not well established. Furthermore, while OAT1 and OAT3 are part of the same subgroup of OATs, other SLC22 organic anion transporters in the kidney fall into a different subgroups based on phylogenetic and functional analysis [19].

With an appreciation of these “operational” considerations, our aim was to combine chemoinformatic analysis of wet lab data with machine learning and deep learning to define sets of molecular properties of relatively small negatively charged drugs that target them for liver (OATP-mediated) versus kidney (OAT-mediated) uptake. This approach was hypothesized to capture the molecular basis of organic anionic small-molecule drugs handled by the liver or the kidney as well as provide a rational basis for understanding the physiochemical properties of drugs that could lead, at the transporter level, to DDI and drug–metabolite interactions. This type of distinction could also be very useful in designing drugs targeting the liver or the kidney or the elimination pathways in which these organs play essential roles.

Although the purpose of these analyses was to define the molecular properties of organic anion drugs taken up by liver OATPs versus the kidney OATs, we note here a general problem in the field is the fact that there is much more quantitative data on drug inhibition of transporter-mediated uptake (i.e., K_i_ data, IC_50_ data) than there is of actual transport of the drug itself (i.e., K_m_ data). Thus, we rely here on measured K_i_ data or calculated K_i_ data (based on IC_50_) with the understanding that it does not necessarily actual reflect transport. On the other hand, it is likely to be a reasonable reflection of the initial interaction with the transporter binding pocket that ultimately can lead to transport, and this interaction may be highly relevant for understanding DDI and drug–metabolite interactions (DMI).

The following sections describe how, beginning with chemoinformatic analyses of over 30 molecular properties of organic anion drugs, we were able to identify eight molecular properties that, together, gave excellent classification of drugs interacting with the liver uptake transporters (liver OATPs) compared to the kidney uptake transporters (kidney OATs). Relative feature importance depended upon the method chosen (e.g., information gain, random forest, FreeViz), and only some of the analysis is presented. Further analysis was done using machine learning and statistical software (Orange, JMP) as well as scripting in the python packages for machine learning (scikit-learn) and neural network building (Keras). Although multiple rounds of (re)analysis were performed, we began with data visualization and statistical analysis, followed by machine learning. Thus, the results are presented in that order.

### 3.2. Chemoinformatic Analysis of Molecular Properties

From the list of over 80 drugs reported to interact selectively with OATP1B1 and/or OATP1B3 or OAT1 and/or OAT3 (Supplementary Materials Table S1), we focused upon a list of over 30 molecular and physiochemical properties of these drugs (Supplementary Materials Table S3). This list of molecular properties, in turn, was derived from larger set of over 70 molecular properties obtained from evaluation of drug structures using the chemoinformatic tools in ICM Chemist Pro. (Complexity values were obtained from PubChem).

From the raw dataset, a standard procedure for data processing was implemented to prepare the input dataset for implementation in JMP statistical software, Orange machine learning software, and various python packages implemented in a jupyter notebook (e.g., pandas, matplotlib, seaborn, scikit-learn). This allowed us to analyze the molecular properties (features) of the drugs (instances) from multiple perspectives. Prior to implementing machine learning and neural network/deep-learning approaches described below, we followed a variety of data visualization, statistical and informational methods to narrow down the key properties distinguishing liver OATPs and kidney OATs.

**Figure 1:**
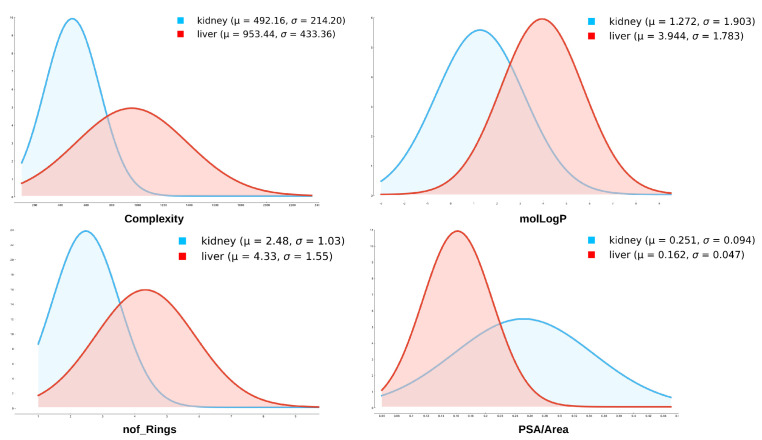
Distribution plots of molecular properties of small organic anion drugs interacting with liver OATPs (SLCO1B1, SLCO1B3, or both) versus kidney OATs (SLC22A6, SLC22A8, or both). Distributions of several molecular properties were analyzed for liver OATPs and kidney OATs. Ultimately, the following molecular properties were chosen: molLogP, PSA/Area, Complexity, nof_Rings, nof_OH, nof_COOH, nof_RotB, and nof_Fragments.

**Figure 2:**
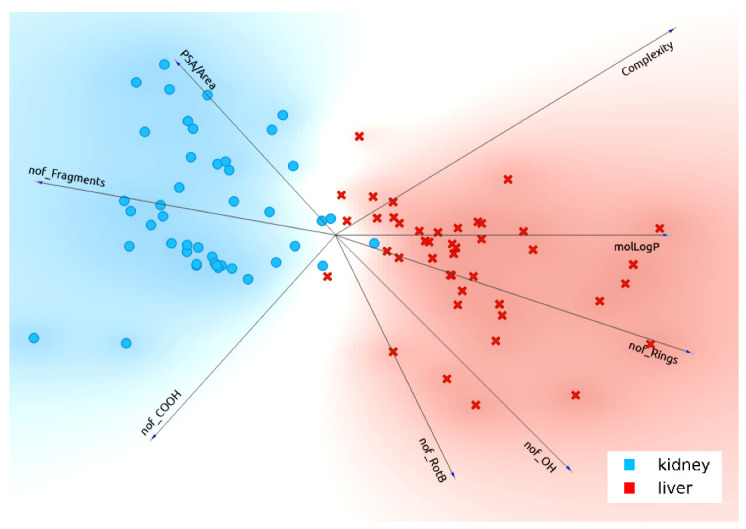
FreeViz diagram segregating kidney and liver drugs based on selected molecular features. FreeViz diagram is one method to analyze the likely importance of the chosen features for determining the interaction of drugs with liver OATPs (Complexity, molLogP, nof_Rings, nof_OH, nof_RotB) versus kidney OATs (nof_COOH, nof_Fragments, PSA/Area). In the figure, length of the arrow reflects the magnitude of the feature according to FreeViz optimization method, and angle between the arrows reflects the correlation between features.

**Figure 3:**
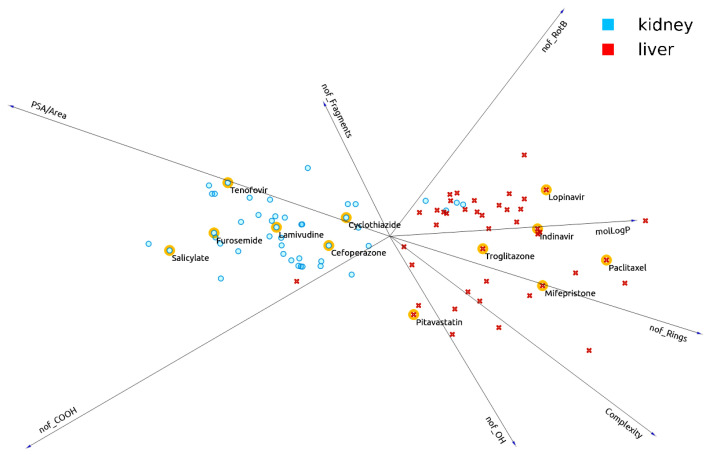
12 drugs with a propensity for interaction with either kidney OATs or liver OATPs highlighted in FreeViz diagram. Quantitative information for the eight molecular properties pertaining to each drug are shown in Table 2.

**Figure 4:**
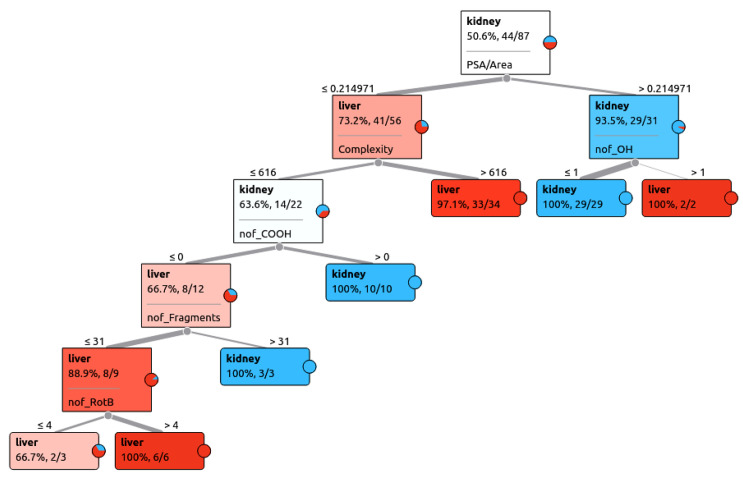
Decision tree used to classify drug interaction with liver OATPs vs kidney OATs based on chosen features. An example of one of the several decision trees generated is shown above. A few of the terminal nodes were examined to determine whether these pure or almost pure nodes contained chemically similar pharmaceuticals as measured by Tanimoto similarity (Supplementary Materials Figure S2).

Many visualizations and spreadsheet analyses were performed, and only a few of the more relevant for understanding the machine learning and neural network analysis are described here. From the plots of drugs interacting with liver OATPs versus kidney OATs (Figure 1), it is apparent that PSA/Area for drugs interacting with liver OATPs differ from kidney OATs. PSA/Area is a surface-normalized feature that is reflective of the density of surface groups capable of hydrogen bonding. Consistent with this, in the drug dataset, it positively correlates with molLogS (r ≈ 0.4), a measure of aqueous solubility, and negatively correlates with molLogP (r ≈−0.7), a measure of lipid solubility. PSA/Area of drugs interacting with liver OATPs is lower than for drugs interacting with kidney OATs. Such differences between drugs interacting with liver OATPs versus kidney OATs can also be seen with Complexity and nof_Rings, with drugs favoring liver OATPs having both a higher Complexity and greater number of ringed structures. Thus, the exploratory data analysis suggested PSA/Area, Complexity, nof_Rings, and molLogP may be key properties distinguishing drugs interacting with liver OATPs and kidney OATs. Nevertheless, in the univariate analyses, one finds some overlap between OATs and OATPs. However, when multiple molecular properties are considered, as in the FreeViz diagrams and decision tree classification, there is much better separation between kidney OAT-interacting drugs and liver OATP-interacting drugs. (This is discussed below in the context of Figure 2, Figure 3 and Figure 4).

### 3.3. Selection of Molecular Properties (Feature Selection) for Machine Learning Classification of Liver OATP Drugs and Kidney OAT Drugs

In an integrative approach towards feature selection, we also employed JMP’s feature predictor screening tool which uses a bootstrap forest approach (data not shown). Orange allowed evaluation of information gain for each feature. Since the aforementioned feature screening methodologies use different algorithms, they may favor different feature sets. To help resolve this issue, certain highly correlated features (e.g., molArea, molVolume, molWeight) were eliminated in favor of a single one that captured multiple aspects of the removed features and possibly some other key aspect of the structure (Supplementary Materials Figure S1). For example, Complexity correlated very highly with molWeight (r > 0.9). Since we needed a limited set of features (ideally less than one-tenth of the total number of instances), we chose eight features (Table 1) based on distribution plots and visualizations, aforementioned feature selection algorithms, and interpretability. These were: PSA/Area, molLogP, Complexity, nof_Rings, nof_OH, nof_COOH, nof_Fragments, and nof_RotB. By principal components analysis, this feature set accounted for around 75–80% of variance in the data (three components).

The FreeViz diagram (Figure 2 and Figure 3) is another way of evaluating the potential importance of the selected features for classifying drugs preferring liver OATPs versus kidney OATs. FreeViz is an optimization method in Orange that analyzes the importance and relationship of features resulting in a circular diagram that in this case, shows what features (molecular properties) in drugs predispose to interaction with the liver OATPs as compared to the kidney OATs. In these figures, the angle between the arrows is related to the correlations between various features, and the magnitude is related to the potential contribution of each feature. Through FreeViz, these eight features appear to separate the liver OATPs from the kidney OATs and thus support the feature selection strategy described above. Although a few of the molecular properties are correlated, we considered it important to retain them in light of the other analyses already described and their interpretability in the binary classification using machine learning and neural networks described below.

**Table 2:** Eight molecular properties for 12 drugs highlighted in the FreeViz diagram (Figure 3).

Drug	PubChemID	Organ	nof_OH	nof_Fragments	molLogP	nof_RotB	nof_Rings	nof_COOH	Complexity	PSA/Area
Tenofovir	25448811	Kidney	0	19	−2.9691	5	2	0	354	0.383860
Furosemide	10991988	Kidney	0	32	1.5688	5	2	1	481	0.328760
Salicylate	17553798	Kidney	0	15	1.2562	1	1	1	133	0.318860
Cefoperazone	15618660	Kidney	0	45	−0.3194	12	5	1	1250	0.299100
Cyclothiazide	25448811	Kidney	0	40	1.3583	2	4	0	758	0.309350
Lamivudine	18174163	Kidney	1	15	−1.0496	2	2	0	331	0.317660
Pitavastatin	16595711	Liver	3	31	4.6637	8	4	1	631	0.162070
Lopinavir	20102298	Liver	1	46	5.2453	17	4	0	940	0.133870
Indinavir	20102298	Liver	2	45	2.8412	14	5	0	952	0.139510
Paclitaxel	18321482	Liver	3	64	3.3763	15	7	0	1790	0.201475
Mifepristone	18321482	Liver	1	34	5.2420	3	5	0	921	0.065822
Troglitazone	18321482	Liver	1	31	4.6699	5	4	0	681	0.145090

### 3.4. Effectiveness of Set of 8 Molecular Properties in Machine Learning Analysis of Drugs Preferring Liver OATPs versus Kidney OATs

Machine learning and neural network analysis was done using Orange, JMP, and python packages. Orange is an open-source machine learning and data visualization software; JMP is a statistical and data analytics package that interfaces with SAS. Much of the initial analysis was done with Orange and JMP. Machine learning scripts were also written in python using the jupyter notebook. Libraries such as pandas, numpy, matplotlib, and seaborn were imported for data preparation, analysis, and visualization. Generally, scikit-learn and Keras were used, respectively, to perform machine learning (e.g., random forest classification, logistic regression, decision tree classification, k-nearest neighbors classification) or to build neural networks (e.g., sequential model). In general, these python-based codes were used to confirm the results obtained in Orange and to assess the impact of choosing different parameters (as opposed to default choices) for individual classification algorithms.

As can be seen in Table 3, with the chosen set of eight features (molecular properties), binary classification accuracies in range of ≈75–99% (over many runs using the “leave-one-out” method in Orange test and score) were obtained depending on the ML approach employed. This method included k-nearest neighbors, decision tree, random forests, logistic regression, and naïve bayes approaches. Area under ROC curves (AUC) were also high, in the range of 0.9 or above (Table 3). Given the high classification accuracies, it is not surprising that the number of misclassified instances were low.

With a decision tree (Figure 4), it is often easy to appreciate the role different features (in this case, molecular properties of drugs) in the classification of drugs interacting with liver OATPs versus kidney OATs; in other words, how an algorithm “thinks” as it reduces entropy (disorder in data) at each decision node by evaluating information gain.

Decision tree classification accuracies were generally less than for random forests but still in the 80–95% range, depending on the run. In the decision tree shown, most important features for classification were PSA/Area, Complexity, nof_OH, nof_COOH, nof_fragments, and nof_RotB. In other decision trees, it was interesting to note that even though the homogenous terminal node in the tree arrived through a series of binary decisions involving several molecular properties, within those nodes, the chemical similarity of drugs, as measured by the Tanimoto similarity score, was modest (Supplementary Materials Figure S2). Thus, it appears, at least in this analysis of liver OATPs versus kidney OAT-interacting drugs, sets of molecular properties which eventually result in a pure terminal node on the decision tree do not necessarily correspond to drugs with high chemical similarity as measured by Tanimoto similarity score.

### 3.5. Comparison of Deep-Learning Models with Other Machine Learning Models

**Figure 5:**
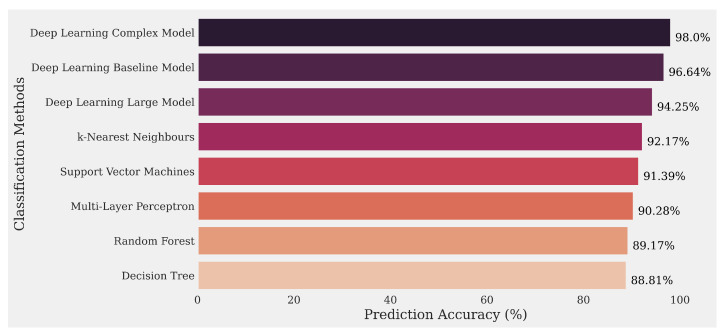
Bar plot representing the cross-validation classification accuracies for different deep learning and machine learning models. Description of each model and its associated property are mentioned in Table 4.

Three deep-learning models were prepared to test the accuracy of the selected features for classifying the OAT-interacting drugs and the OATP-interacting drugs correctly. Keras, a deep-learning Artificial Programming Interface (API) built on top of Tensorflow, an open-source machine learning library, was used to prepare these models. These models are designated as the “baseline”, “large”, or “complex” model depending on the number of nodes and number of the hidden layers as well the neural network’s activation functions. For example, the complex model has three hidden layers and employs the rectified linear unit (ReLU) and the sigmoid activation functions. In contrast, the baseline model has just one hidden layer with the ReLU activation function. From the accuracy plot (Figure 5), it is evident that deep-learning models performed better than any other machine learning model coded using the scikit-learn library using the same feature set. These models included the multilayer perceptron (MLP), a feedforward artificial neural network model that was used to determine the classification accuracy. Other ML models such as random forest, k-nearest neighbors, decision tree, and support vector machines were also implemented. Although deep-learning models and MLP performed exceptionally well, other machine learning models also had very high accuracies (Classification accuracies of scikit-learn ML models are slightly different from Table 3 because those results were obtained using the Orange data mining software, generally with default settings and using the “leave-one-out” method).

## 4. Discussion

At the range of pH values in patients who are administered pharmaceutical drugs, many drugs are small negatively charged organic molecules with molecular masses roughly in the range of 150 kDa to 1200 kDa [13,46]. Such molecules, which include not only drugs, but endogenous metabolites, nutrients, toxins, gut microbe products, and signaling molecules fall into a general category referred to as organic anions. In the case of drugs, after entering the bloodstream, the organic anions, which are often protein (albumin)-bound, are eventually taken up through “uptake SLC transporters” into various organs [7].

From the perspective of “drug ADME” (absorption, distribution, metabolism, and excretion), the most important organs for metabolism and excretion are the liver and kidney [13,14,50]. In hepatocytes, the key drug uptake transporters are generally held to be the multi-specific transporters known as organic anion transporter polypeptides, or OATPs (OATP1B1 or SLCO1B1; OATP1B3 or SLCO1B3), while in the kidney, the relevant multi-specific transporters are the organic anion transporters, or OATs (OAT1 or SLC22A6; OAT3 or SLC22A8). These are four of the original “FDA seven transporters”; because of their pharmacokinetic importance, new drug entities must be evaluated for transport by these transporters [16]. Apart from the large amount of *in vitro* transport and binding data for these transporters, there is a considerable amount of data from knockout mice supporting in vivo roles in drug, toxin, and metabolite handling [51,52,53,54,55,56,57,58,59,60,61,62,63,64].

Because OATP1B1 and OATP1B3 are highly expressed in the liver but not the kidney, and OAT1 and OAT3 are found to be highly expressed in the kidney but minimally if at all in the liver and because OATPs are thought to account for most liver uptake of organic anion drugs and likewise with OATs in the kidney, it is reasonable to roughly approximate hepatic organic anion drug uptake by considering liver OATPs together and likewise by considering the main renal OATs together. As described elsewhere in this article, we recognize that there are other transporters of organic anions in the liver and kidney, but the evidence in the literature strongly supports the “operational view” that the SLC transporters we have considered to be liver OATPs and kidney OATs make a very substantial contribution to hepatic and renal uptake of small-molecule drugs that are organic anions.

Chemoinformatic analysis of molecular properties of small organic molecules, together with ML/deep-learning methods, have been shown to be effective in distinguishing preferences for one or another multi-specific drug transporter [43,46]. Applying a similar approach to liver/OATP-transported drugs versus kidney/OAT-transported drugs can yield a deeper understanding of molecular properties important for hepatic versus renal uptake. More importantly, these molecular properties can be useful for understanding pharmacokinetics of drugs and drug combinations and for physiologically based pharmacokinetic (PBPK) modeling [14]. Engineering or optimizing molecular properties of drugs that prefer hepatic OATP uptake over renal OAT uptake also provides a basis for tissue targeting to the liver versus the kidney. In the setting of moderate or severe liver or kidney disease, such an understanding could be critical for optimizing drug treatment, especially when multiple options are available. For example, antivirals that are used for HIV treatment, or have been considered to be treatment for COVID-19, include both those with primary (or initial) OAT-mediated renal uptake or OATP-mediated hepatic uptake [65]. In both viral diseases, setting of organ failure is not uncommon. For instance, patients with HIV or COVID-19 frequently have some degree of kidney disease. Assuming a choice of equally effective antivirals transported by liver OATPs vs kidney OATs, a clinician may consider the use of the liver OATP-transported drug in the setting of chronic kidney disease.

By analyzing individual molecular properties using data visualization techniques (e.g., distributions, boxplots), as well as statistical and information-based approaches to evaluate feature importance (e.g., bootstrap forest predictor screening, information gain), we narrowed down >30 molecular properties of drugs to eight properties. These eight molecular properties (features): PSA/Area, molLogP, Complexity, nof_Rings, nof_OH, nof_COOH, nof_Fragments, and nof_RotB were sufficient to achieve 75% to 99% accuracy for classifying drugs preferring “liver OATPs” versus “kidney OATs” using machine learning (e.g., random forest, logistic regression, decision tree) and deep-learning algorithms. Although it is often difficult to evaluate which features are most important in a classification method (e.g., k-nearest neighbors) and while the relative importance of individual features varies from method to method, and while it is particularly difficult to assess the importance of feature combinations in classification, we can make some general points after considering the features from the several viewpoints described in the Results.

In general, liver OATPs prefer compounds with higher molLogP (more hydrophobicity), a larger number of ringed structures, and greater Complexity. In contrast, OATs prefer to interact with more polar drugs (higher PSA/Area) and drugs with more carboxyl groups. These points are illustrated in the FreeViz optimization (Figure 2) and decision tree (Figure 4). The relative importance of other features (i.e., number of OH groups, number of fragments, number of rotatable bonds) can also be appreciated (Figure 1, Figure 2 and Figure 3, Table 2). It is worth emphasizing that while OAT1-interacting drugs were reasonably well separated from OATP-interacting drugs by many univariate analyses of individual molecular properties, OAT3-interacting drugs sometimes overlapped slightly with OATP-interacting drugs owing to their higher hydrophobicity, greater number of ringed structures, and higher Complexity. This may explain why, in our initial attempts at classification, OAT3-interacting drugs were sometimes misclassified as OATPs in confusion matrices. As classification accuracies improved with feature set refinement and, to some extent, algorithm parameters, such that accuracies of 85% to >95% were obtained, the number of misclassified drugs became so small that it was difficult to assess whether OAT3 drugs were more commonly misclassified as OATP drugs.

Because many frequently prescribed drugs such as antibiotics, NSAIDs, antivirals, statins used for hypercholesterolemia, a variety of antihypertensive drugs, and chemotherapeutic agents are taken up through either the liver OATPs or the kidney OATs, the results of our studies are useful for understanding and predicting potential DDI. It is important to consider such DDI at the level of individual transporters i.e., OATP1B1 (SLCO1B1), OATP1B3 (SLCO1B3), OAT1 (SLC22A6), and OAT3 (SLC22A8) and at the level of sets of transporters. Especially when potentially highly toxic chemotherapeutic agents are involved, it is worth considering what other drugs the patient has been prescribed that use the same transporter or set of transporters. For example, for mild to moderate hypertension there are many therapeutic options. The patient can also be advised about over-the-counter medications, such as analgesics that may compete for binding to the transporter. Using chemoinformatic analyses together with ML/deep learning, we present a formal basis, grounded in a set of molecular and physiochemical properties of the drugs themselves, for approaching these issues.

Drug–metabolite interactions are an area of considerable clinical concern. OATs and OATPs are now known to play pivotal roles in regulating in vivo metabolism and other aspects of systemic physiology through their transport of metabolites (e.g., TCA cycle intermediates), signaling molecules (e.g., cyclic nucleotides, prostaglandins, short-chain fatty acids), hormones (e.g., thyroxine), vitamins (e.g., pantothenic acid), and gut microbiome products (e.g., tryptophan metabolites, secondary bile acids) [29,33,43,61,62,64,66,67]. Drugs can compete for transport of these metabolites, raising the possibility of metabolic derangements due to drug–metabolite interactions [14]. This is an area of concern in, for example, drug treatment during pregnancy [68,69].

It is also a major concern in disease states where metabolism is already perturbed, and elimination of drugs and metabolites compromised. For example, the uremic solutes and uremic toxins in chronic kidney disease, where many gut microbe-derived products act as uremic toxins or precursors to uremic toxins that are associated with cell and tissue dysfunction and damage [30,36,70]. Many of these appear to be primarily OAT substrates in vivo and depend on proper renal tubular function for elimination [64]. In this regard, it is worth noting that a chemoinformatics-machine learning approach has been used to classify OAT1 versus OAT3 metabolites that are uniquely elevated in OAT1 and OAT3 knockout mice [43].

Our study is limited by the fact that we used FDA-approved drugs. These drugs have, of course, made it through animal and human testing and are often administered in pill form. Thus, there is a bias both toward drugs that passed safety criteria and made it to market and those which are reasonably well absorbed through the gastrointestinal tract. However, these are also largely the drugs that have been carefully analyzed in in vitro transport assays. In the future, it would be important to use a larger, unbiased (or less biased) set of organic anion molecules that includes not only drugs but also metabolites, natural products, and toxins—assuming there are sufficient numbers that have been carefully studied for transport by OATs and OATPs. Furthermore, while these drugs appear to act as organic anions under conditions of in vitro assays, in some cases their charge could be different in vivo—especially when pH varies. However, we note that the eight molecular properties we used are, for the most part, independent of charge.

Although a sufficient number of OATP1B1 and OATP1B3 endogenous metabolites have not been established in vivo (e.g., through more comprehensive metabolomics analysis of OATP1B1 and OATP1B3 knockout mice), it should eventually be possible to use the strategy we have employed here, based on molecular properties of the small molecules themselves, to formally predict likely drug–metabolite interactions (DMI). Such predictions can then be used to plan studies to assess DMI in normal humans and patients with diseases such as CKD, liver disease, metabolic syndrome, and diabetes. Combined chemoinformatics-machine learning approaches should also prove useful for repositioning drugs with existing indications for new indications [71,72]. The types of methods employed here will be an essential step toward more rational treatment with drugs or drug combinations and for decreasing the chance of metabolic imbalances or worsening of metabolic diseases.

## Figures and Tables

**Table 1:** Final set of eight features chosen for machine learning analysis.

Feature	Description
nof_OH	Number of hydroxyl (OH) groups in the molecule
nof_Fragments	Possible number of molecular fragments in the molecule
molLogP	Log of lipophilicity (P) of the molecule
nof_RotB	Number of rotatable bonds in the molecule
nof_Rings	Number of rings in the molecule
nof_COOH	Number of carboxyl (COOH) groups in the molecule
Complexity	Molecular complexity
PSA/Area	Polar Surface Area (PSA)/Molecular Area of the molecule

**Table 3:** Table showing the success of different algorithms based on classification accuracy and other metrics. Table above indicates several classification metrics relating to logistic regression, decision tree, random forest, naïve bayes, k-nearest neighbors, the “out of the box” neural network in Orange. These results are reflective of many runs, which generally give a classification accuracy of about 75–99%. Please note that both the classification accuracy and the area under the (ROC) curve are high for nearly all methods. Misclassifications in the confusion matrices were generally low.

Classification	Accuracy	AUC	F1 Score	Precision	Recall
Simple Neural Network	0.954	0.996	0.954	0.955	0.954
Logistic Regression	0.988	0.984	0.988	0.989	0.988
Random Forest	0.885	0.964	0.885	0.885	0.885
Naïve Bayes	0.908	0.949	0.908	0.909	0.908
K-Nearest Neighbors	0.759	0.833	0.758	0.760	0.759
Decision Tree	0.851	0.847	0.851	0.851	0.851

**Table 4:** Deep learning and machine learning models with their properties.

Model	Properties
Deep-Learning “Complex” Model	hidden layers: 3, activation functions: ReLU & sigmoid
Deep-Learning “Baseline” Model	hidden layers: 1, activation function: ReLU
Deep-Learning “Large” Model	hidden layers: 2, activation functions: ReLU & sigmoid
k-Nearest Neighbors	nearest neighbors: 3
Support Vector Machine	kernel: radial basis function
Multilayer Perceptron	hidden layers: 1
Random Forest	estimators: 20
Decision Tree	maximum depth: 4

## Data Availability

See Supplemental Data.

## Supplementary Materials

The following are available online at https://www.mdpi.com/article/10.3390/pharmaceutics13101720/s1, Table S1: List of drugs and their transporters, Table S2: Articles from which Ki data is obtained, Table S3: List of > 30 molecular features used and their description, Figure S1: Correlation Heatmap: Graphical representation of correlation matrix representing correlation between different features, Figure S2: Heat map of selected nodes corresponding to the decision tree (Figure 4), Figure S3: Diagram of a neural network corresponding to Figure 5.
