# Supplementary material for: Molecular Properties of Drugs Handled by Kidney OATs and Liver OATPs Revealed by Chemoinformatics and Machine Learning: Implications for Kidney and Liver Disease
Source: Pharmaceutics. 2021 Oct 18;13(10):1720. doi: 10.3390/pharmaceutics13101720 (PMC8538396; doi:10.3390/pharmaceutics13101720)
Supplement: Supplementary file 1 [file pharmaceutics-13-01720-s001.zip › pharmaceutics-1420552-supplementary.pdf]

Supporting Materials : Molecular Properties of Drugs Handled by Kidney OATs and Liver OATPs Revealed by Chemoinformatics and Machine Learning: *Implications for Kidney and Liver Disease*

Anisha K. Nigam [1,‡], Anupam A. Ojha [2,‡], Julia G. Li [3], Da Shi [1], Vibha Bhatnagar [4], Kabir B. Nigam [6], Ruben Abagyan [1,*] and Sanjay K. Nigam [5,*]

[1] Skaggs School of Pharmacy, University of California San Diego, La Jolla, CA 92093, U.S.
[2] Department of Chemistry and Biochemistry, University of California San Diego, La Jolla, CA 92093, U.S.
[3] Department of Biology, University of California San Diego, La Jolla, CA 92093, U.S.
[4] Department of Family and Preventative Medicine, University of California San Diego, La Jolla, CA 92093, U.S.
[5] Departments of Pediatrics and Medicine (Nephrology), University of California San Diego, La Jolla, CA 92093, U.S.
[6] Virginia Commonwealth University School of Medicine, Richmond, VA 23298, U.S.
* Correspondence: ruben@ucsd.edu (R.A.); snigam@ucsd.edu (S.K.N)
‡ These authors contributed equally to this work

Table S1. List of drugs and their transporters

Sl. No.	Drug Name	Drug Transporter
1	Candesartan	OAT1 and/or OAT3 ($k_i < 250\ \mu$M)
2	Ethacrynate	OAT1 and/or OAT3 ($k_i < 250\ \mu$M)
3	Bumetanide	OAT1 and/or OAT3 ($k_i < 250\ \mu$M)
4	Mycophenolic acid	OAT1 and/or OAT3 ($k_i < 250\ \mu$M)
5	Losartan	OAT1 and/or OAT3 ($k_i < 250\ \mu$M)
6	Furosemide	OAT1 and/or OAT3 ($k_i < 250\ \mu$M)
7	Diclofenac	OAT1 and/or OAT3 ($k_i < 250\ \mu$M)
8	Piroxicam	OAT1 and/or OAT3 ($k_i < 250\ \mu$M)
9	Ketoprofen	OAT1 and/or OAT3 ($k_i < 250\ \mu$M)
10	Sulindac	OAT1 and/or OAT3 ($k_i < 250\ \mu$M)
11	Phenylbutazone	OAT1 and/or OAT3 ($k_i < 250\ \mu$M)
12	Cefadroxil	OAT1 and/or OAT3 ($k_i < 250\ \mu$M)
13	Tolmetin	OAT1 and/or OAT3 ($k_i < 250\ \mu$M)
14	Bendroflumethiazide	OAT1 and/or OAT3 ($k_i < 250\ \mu$M)
15	Naproxen	OAT1 and/or OAT3 ($k_i < 250\ \mu$M)
16	Phenacetin	OAT1 and/or OAT3 ($k_i < 250\ \mu$M)
17	Cyclothiazide	OAT1 and/or OAT3 ($k_i < 250\ \mu$M)
18	Simvastatin	OAT1 and/or OAT3 ($k_i < 250\ \mu$M)
19	Buspirone	OAT1 and/or OAT3 ($k_i < 250\ \mu$M)
20	Mefenamic acid	OAT1 and/or OAT3 ($k_i < 250\ \mu$M)
21	Cefamandole	OAT1 and/or OAT3 ($k_i < 250\ \mu$M)
22	Cephalothin	OAT1 and/or OAT3 ($k_i < 250\ \mu$M)
23	Betamipron	OAT1 and/or OAT3 ($k_i < 250\ \mu$M)
24	Chlorothiazide	OAT1 and/or OAT3 ($k_i < 250\ \mu$M)
25	Trichloromethiazide	OAT1 and/or OAT3 ($k_i < 250\ \mu$M)
26	Cimetidine	OAT1 and/or OAT3 ($k_i < 250\ \mu$M)
27	Methazolamide	OAT1 and/or OAT3 ($k_i < 250\ \mu$M)
28	Didanosine	OAT1 and/or OAT3 ($k_i < 250\ \mu$M)
29	Cefaclor	OAT1 and/or OAT3 ($k_i < 250\ \mu$M)
30	Zalcitabine	OAT1 and/or OAT3 ($k_i < 250\ \mu$M)
31	Lamivudine	OAT1 and/or OAT3 ($k_i < 250\ \mu$M)
32	Loxoprofen	OAT1 and/or OAT3 ($k_i < 250\ \mu$M)
33	Cefotiam	OAT1 and/or OAT3 ($k_i < 250\ \mu$M)
34	Cilastatin	OAT1 and/or OAT3 ($k_i < 250\ \mu$M)
35	Etodolac	OAT1 and/or OAT3 ($k_i < 250\ \mu$M)
36	Salicylate	OAT1 and/or OAT3 ($k_i < 250\ \mu$M)
37	Cefoperazone	OAT1 and/or OAT3 ($k_i < 250\ \mu$M)
38	Cefotaxime	OAT1 and/or OAT3 ($k_i < 250\ \mu$M)
39	Hydrochlorothiazide	OAT1 and/or OAT3 ($k_i < 250\ \mu$M)
40	Stavudine	OAT1 and/or OAT3 ($k_i < 250\ \mu$M)
41	Zidovudine	OAT1 and/or OAT3 ($k_i < 250\ \mu$M)
42	Mercaptopurine	OAT1 and/or OAT3 ($k_i < 250\ \mu$M)
43	Temocaprilat	OAT1 and/or OAT3 ($k_i < 250\ \mu$M)
44	Tenofovir	OAT1 and/or OAT3 ($k_i < 250\ \mu$M)

Sl. No.	Drug Name	Drug Transporter
45	Rosiglitazone	OATP1B1 and/or OATP1B3 ($k_i < 250\ \mu M$)
46	Paclitaxel	OATP1B1 and/or OATP1B3 ($k_i < 250\ \mu M$)
47	Venetoclax	OATP1B1 and/or OATP1B3 ($k_i < 250\ \mu M$)
48	Vincristine	OATP1B1 and/or OATP1B3 ($k_i < 250\ \mu M$)
49	Rilpivirine	OATP1B1 and/or OATP1B3 ($k_i < 250\ \mu M$)
50	Darunavir	OATP1B1 and/or OATP1B3 ($k_i < 250\ \mu M$)
51	Repaglinide	OATP1B1 and/or OATP1B3 ($k_i < 250\ \mu M$)
52	Carbamazepine	OATP1B1 and/or OATP1B3 ($k_i < 250\ \mu M$)
53	Amprenavir	OATP1B1 and/or OATP1B3 ($k_i < 250\ \mu M$)
54	Lapatinib	OATP1B1 and/or OATP1B3 ($k_i < 250\ \mu M$)
55	Bosutinib	OATP1B1 and/or OATP1B3 ($k_i < 250\ \mu M$)
56	Telithromycin	OATP1B1 and/or OATP1B3 ($k_i < 250\ \mu M$)
57	Afatinib	OATP1B1 and/or OATP1B3 ($k_i < 250\ \mu M$)
58	Sorafenib	OATP1B1 and/or OATP1B3 ($k_i < 250\ \mu M$)
59	Neratinib	OATP1B1 and/or OATP1B3 ($k_i < 250\ \mu M$)
60	Nilotinib	OATP1B1 and/or OATP1B3 ($k_i < 250\ \mu M$)
61	Vandetanib	OATP1B1 and/or OATP1B3 ($k_i < 250\ \mu M$)
62	Sunitinib	OATP1B1 and/or OATP1B3 ($k_i < 250\ \mu M$)
63	Gefitinib	OATP1B1 and/or OATP1B3 ($k_i < 250\ \mu M$)
64	Montelukast	OATP1B1 and/or OATP1B3 ($k_i < 250\ \mu M$)
65	Atazanavir	OATP1B1 and/or OATP1B3 ($k_i < 250\ \mu M$)
66	Fusidic Acid	OATP1B1 and/or OATP1B3 ($k_i < 250\ \mu M$)
67	Sildenafil	OATP1B1 and/or OATP1B3 ($k_i < 250\ \mu M$)
68	Grazoprevir	OATP1B1 and/or OATP1B3 ($k_i < 250\ \mu M$)
69	Lopinavir	OATP1B1 and/or OATP1B3 ($k_i < 250\ \mu M$)
70	Rifamycin SV	OATP1B1 and/or OATP1B3 ($k_i < 250\ \mu M$)
71	Cyclosporine	OATP1B1 and/or OATP1B3 ($k_i < 250\ \mu M$)
72	Sulfasalazine	OATP1B1 and/or OATP1B3 ($k_i < 250\ \mu M$)
73	Troglitazone	OATP1B1 and/or OATP1B3 ($k_i < 250\ \mu M$)
74	Saquinavir	OATP1B1 and/or OATP1B3 ($k_i < 250\ \mu M$)
75	Mifepristone	OATP1B1 and/or OATP1B3 ($k_i < 250\ \mu M$)
76	Estradiol	OATP1B1 and/or OATP1B3 ($k_i < 250\ \mu M$)
77	Clarithromycin	OATP1B1 and/or OATP1B3 ($k_i < 250\ \mu M$)
78	Erythromycin	OATP1B1 and/or OATP1B3 ($k_i < 250\ \mu M$)
79	Indinavir	OATP1B1 and/or OATP1B3 ($k_i < 250\ \mu M$)
80	Erlotinib	OATP1B1 and/or OATP1B3 ($k_i < 250\ \mu M$)
81	Digoxin	OATP1B1 and/or OATP1B3 ($k_i < 250\ \mu M$)
82	Doxorubicin	OATP1B1 and/or OATP1B3 ($k_i < 250\ \mu M$)
83	Fexofenadine	OATP1B1 and/or OATP1B3 ($k_i < 250\ \mu M$)
84	Bosentan	OATP1B1 and/or OATP1B3 ($k_i < 250\ \mu M$)
85	Cilostazol	OATP1B1 and/or OATP1B3 ($k_i < 250\ \mu M$)
86	Docetaxel	OATP1B1 and/or OATP1B3 ($k_i < 250\ \mu M$)
87	Pitavastatin	OATP1B1 and/or OATP1B3 ($k_i < 250\ \mu M$)

Table S2. Articles from which K_i data is obtained

**CID**	**Drug Name**	**PMID**
CID	Drug Name	PMID
2541	Candesartan	17674156
3278	Ethacrynate	14610216
2471	Bumetanide	11426832; 10991954
446541	Mycophenolic acid	17462604
3961	Losartan	17674156
3440	Furosemide	10991988
3033	Diclofenac	14722319
54676228	Piroxicam	10220563
3825	Ketoprofen	14722319
1548887	Sulindac	14722319
4781	Phenylbutazone	14722319
47965	Cefadroxil	11909604
5509	Tolmetin	14722319
2315	Bendroflumethiazide	18216144
156391	Naproxen	14722319
4754	Phenacetin	10220563
2910	Cyclothiazide	14610216
54454	Simvastatin	14729100
2477	Buspirone	19737926
4044	Mefenamic acid	12388633
456255	Cefamandole	11909604
6024	Cephalothin	12005172
71651	Betamipron	11426832
2720	Chlorothiazide	14610216
5560	Trichloromethiazide	14610216
2756	Cimetidine	14978359
4100	Methazolamide	14610216
50599	Didanosine	18174163
51039	Cefaclor	16098483
24066	Zalcitabine	18174163
60825	Lamivudine	18174163
3965	Loxoprofen	15548848
43708	Cefotiam	16098483
6435415	Cilastatin	11426832
3308	Etodolac	10991954
54675850	Salicylate	17553798
44187	Cefoperazone	15618660
5742673	Cefotaxime	11909604
3639	Hydrochlorothiazide	10991988
18283	Stavudine	18174163
35370	Zidovudine	10945832
667490	Mercaptopurine	15287899
443151	Temocaprilat	12660303

CID	Drug Name	PMID
464205	Tenofovir	25448811
77999	Rosiglitazone	18314419
36314	Paclitaxel	18321482
49846579	Venetoclax	26927160
5978	Vincristine	22541068
6451164	Rilpivirine	23428312
213039	Darunavir	20102298
65981	Repaglinide	18314419
2554	Carbamazepine	18321482
65016	Amprenavir	20102298
208908	Lapatinib	25165131
5328940	Bosutinib	25165131
3002190	Telithromycin	17296622
10184653	Afatinib	25165131
216239	Sorafenib	25165131
9915743	Neratinib	25165131
644241	Nilotinib	25165131
3081361	Vandetanib	25165131
5329102	Sunitinib	25165131
123631	Gefitinib	25165131
5281040	Montelukast	16495352
148192	Atazanavir	20102298
3000226	Fusidic Acid	26888941
135398744	Sildenafil	17496208
44603531	Grazoprevir	29572333
92727	Lopinavir	20102298
6324616	Rifamycin SV	12085361
5284373	Cyclosporine	16495352
5339	Sulfasalazine	22541068
5591	Troglitazone	18321482
441243	Saquinavir	20102298
55245	Mifepristone	18321482
5757	Estradiol	18321482
84029	Clarithromycin	17296622
12560	Erythromycin	17296622
5362440	Indinavir	20102298
176870	Erlotinib	22541068
2724385	Digoxin	20102298
31703	Doxorubicin	22541068
3348	Fexofenadine	16014768
104865	Bosentan	17496208
2754	Cilostazol	28535976
148124	Docetaxel	27452633
5282452	Pitavastatin	16595711

Table S3. List of > 30 molecular features used and their description

Feature	Description
molWeight	Molecular weight
molVolume	Molecular Volume
molLogP	Log of lipophilicity (P) of the molecule
mollLogS	Log of solubility (S) of the molecule
molPSA	Polar Surface Area (PSA) of the molecule
molArea	Molecular surface area
PSA/Area	Polar Surface Area (PSA)/Molecular Area of the molecule
nof_Atoms	Number of atoms in the molecule
nof_Chirals	Number of chiral atoms in the molecule
nof_HBA	Number of hydrogen bond acceptors (HBA) in the molecule
nof_HBD	Number of hydrogen bond donors (HBD) in the molecule
molCharge_total	Overall charge of the molecule
nof_Rings	Number of rings in the molecule
Complexity	Molecular complexity
nof_PosCharge	Number of positive charged atoms in the molecule
nof_negCharge	Number of negative charged atoms in the molecule
a_heavy	Number of heavy atoms in the molecule
C_R2	Number of carbon atoms attached to two alkyl groups in the molecule
C_R1	Number of carbon atoms attached to one alkyl groups in the molecule
C_R0	Number of carbon atoms attached to no alkyl groups in the molecule
C_sp3	Number of sp^3 - hybridised carbon atoms
posCharge/Volume	Positive charge/molecular volume
negCharge/Volume	Negative charge/molecular volume
nof_RotB	Number of rotatable bonds in the molecule
nof_Fragments	Possible number of molecular fragments in the molecule
nof_PO4	Number of phosphate groups (PO_4) in the molecule
nof_SO3H	Number of sulfo groups (SO_3H) in the molecule
nof_SH	Number of thiol groups (SH) in the molecule
nof_NH2	Number of amino groups (NH_2) in the molecule
nof_COOH	Number of carboxyl groups (COOH) in the molecule
nof_OH	Number of hydroxyl groups (OH) in the molecule
nof_acetyl	Number of acetyl groups in the molecule

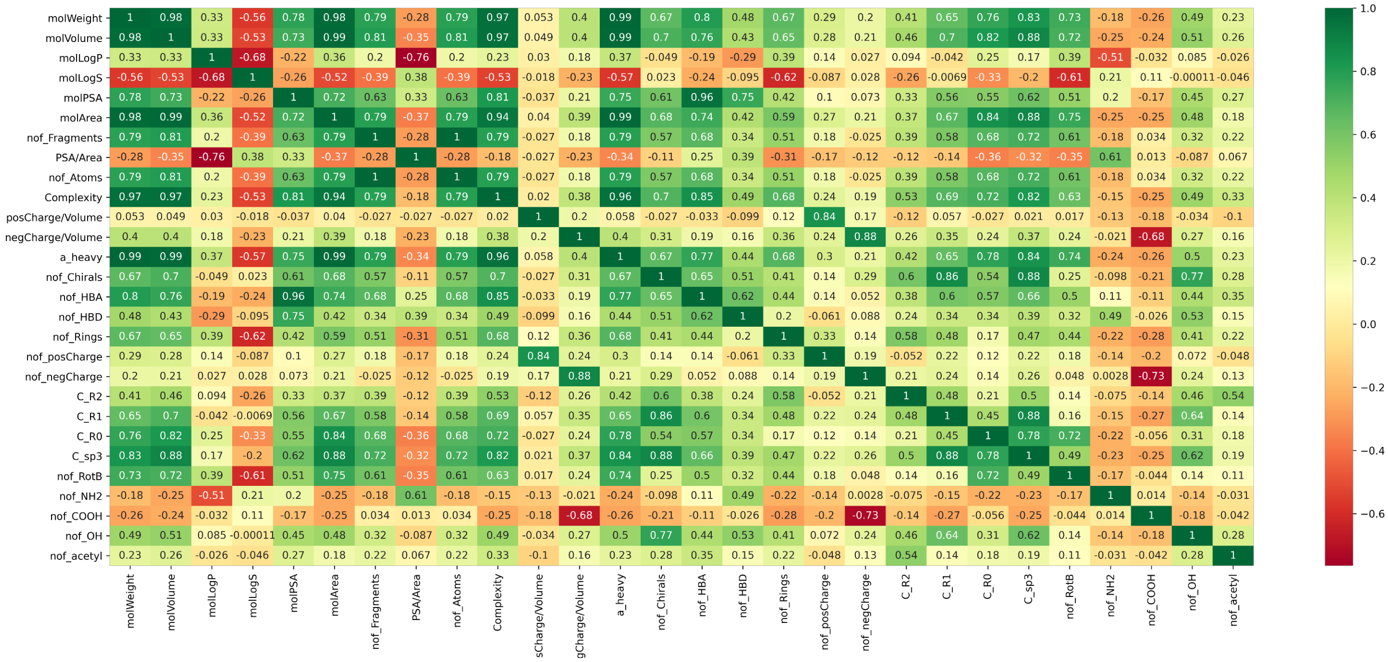

Figure S1. Correlation Heatmap : Graphical representation of correlation matrix representing correlation between different features.

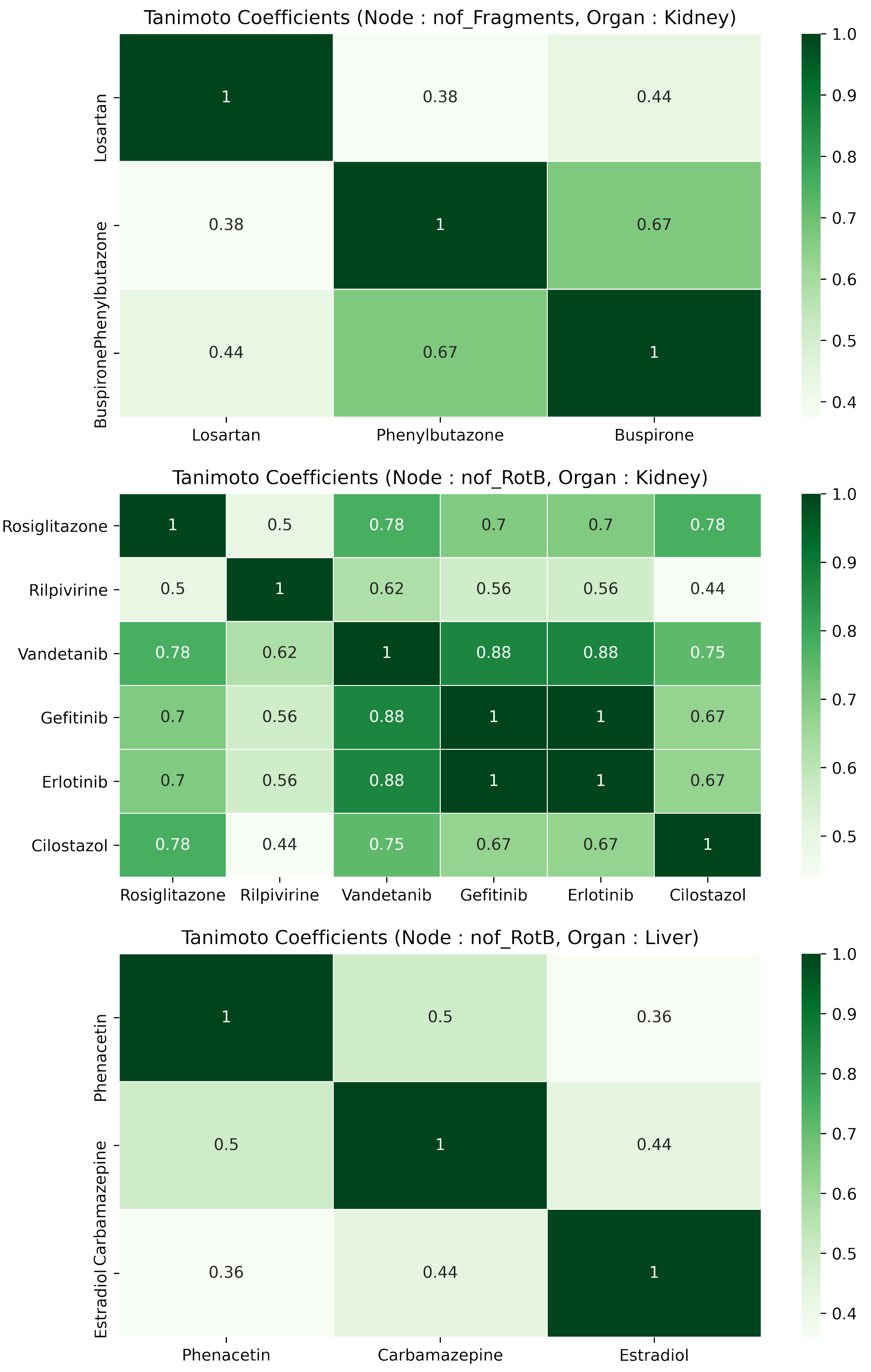

Figure S2. Heat map of selected nodes corresponding to the decision tree (Figure 4). Each heatmap displays the Tanimoto coefficients between drugs from terminal nodes of the decision tree. Nodes were selected from the left-sided branch of the tree after branching on nof_Fragments and nof_RotB.

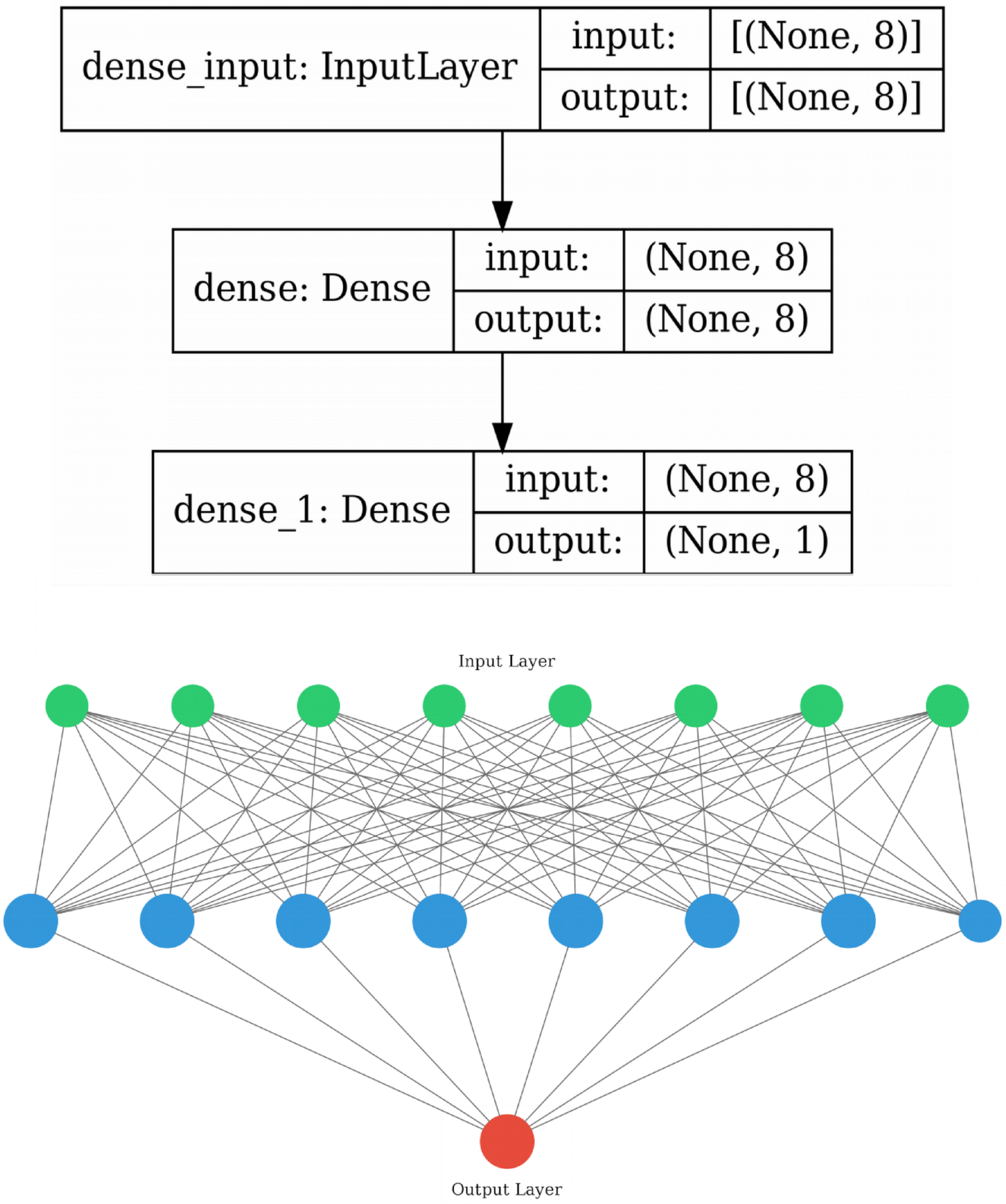

Figure S3. Diagram of a neural network corresponding to Figure 5.
